# Obesity and health service utilization in Brazil: data from the National Health Survey

**DOI:** 10.1186/s12889-022-13906-2

**Published:** 2022-08-02

**Authors:** Karina Abibi Rimes-Dias, Janaina Calu Costa, Daniela Silva Canella

**Affiliations:** 1 Postgraduate Program in Food, Nutrition, and Health, Rio de Janeiro State University, Rio de Janeiro, RJ Brazil; 2 International Center for Equity in Health, Federal University of Pelotas, Pelotas, Rio Grande do Sul, RS Brazil; 3 Institute of Nutrition, Rio de Janeiro State University, Rio de Janeiro, RJ Brazil

**Keywords:** Obesity, Health services, Health surveys, Epidemiology

## Abstract

**Background:**

Obesity, a complex public health problem, is generally associated with other chronic diseases. The association of obesity with health service utilization has been little investigated in low- and middle-income countries. This study aimed to analyze the association between obesity and health service utilization (considering those services related to hypertension and/or diabetes).

**Methods:**

A cross-sectional, nationally-representative, study of Brazilians aged ≥18 years was conducted. Data from the National Health Survey (2013) for 59,402 individuals were analyzed, including measured weight and height. The association between body mass index (BMI) category (under/normal weight, overweight, and obesity) and health service utilization due to hypertension and/or diabetes was investigated using Poisson regression models (crude and adjusted). To analyze the health services utilization, the following variables were considered: 1) routine visits to a general doctor or health service; 2) referrals/consultations with a specialist; 3) prescribed exams done; and 4) hospital admission due to the disease or related complication. All analyses were stratified by sex.

**Results:**

Compared with under/normal-weight individuals, subjects with obesity (both male and female) made roughly double the use of all health care services assessed. Men with hypertension that had obesity had a higher risk of hospital admission (adjusted PR = 2.55; 95%CI 1.81–3.61), than those with under/normal weight. Women with diabetes that had obesity had more referrals/consultations with specialists (adjusted PR = 2.56; 95%CI 1.94–3.38), than those with under/normal weight.

**Conclusions:**

The presence of obesity was associated with increased use of health care services for hypertension and/or diabetes, indicating greater demand for human resources and materials, and a greater burden on the national health system.

**Supplementary Information:**

The online version contains supplementary material available at 10.1186/s12889-022-13906-2.

## Background

Once regarded as a disease-specific to high-income countries, obesity is rising in low and middle-income countries too [1], where are living almost 2 out of every 3 individuals with obesity worldwide [2]. Obesity is a complex chronic disease, defined by World Health Organization (WHO) as “abnormal or excessive fat accumulation that presents a risk to health” [3]. It has multiple determinants and consequences, currently affecting several population groups [4, 5].

On a global level, over 50% of the 693 million individuals with obesity can be found in only 10 countries, including Brazil, which ranks fifth on this list [2]. Between 2006 and 2019, the prevalence of obesity in Brazil rose from 11.8 to 20.3%, an average increase of 0.6% a year. This growth was observed nationwide, in both sexes and across all age groups and educational levels [6].

Obesity stands out for being both a non-communicable disease (NCD) and a risk factor for other NCDs, such as arterial hypertension and diabetes mellitus [4, 7–9]. Hypertension is about twice as prevalent in men and women with obesity as in those with a normal weight [10]. In Brazil, it was estimated that approximately 40.0% of diabetes cases are attributed to obesity, especially among women [11].

Besides being associated with high mortality, chronic NCDs can lead to disabilities [12]. In Brazil, NCDs represent a major public health problem [13]. Rates of hypertension (one of the most important risk factors for cardiovascular diseases) and diabetes are climbing every year, affecting 25.2 and 8.2% of Brazilian adults in 2020, respectively [14].

Given the long-term nature of NCDs, they typically require constant health care, in a generally non-curative process of treatment, that requires routine preventive and therapeutic interventions. Consequently, permanent health system utilization is often necessary for patients with chronic diseases [14].

Health service utilization represents the center of health system functioning, encompassing all direct (consultations, hospitalizations) or indirect (preventive and diagnostic exams) contact with health services [15]. The use of these services is generally associated with a greater need for health care [16], and stems from multiple interacting factors, including individual and contextual aspects, and those related to the type of care [15]. In Brazil, the literature shows greater use of these services by women, individuals with chronic diseases, and patients with a higher number of comorbidities [17–20].

With regard to obesity, despite its growing prevalence in practically all countries, with rates almost trebling over the last four decades [21], this chronic disease appears to be yet invisible in primary health care in Brazil [22]. In addition, there is a dearth of studies on the association between obesity and health service utilization, particularly in low- and middle-income countries. The majority of Brazilian studies investigating the factors related to the use of health services fail to include obesity [16, 20, 23–25], while those addressing the condition have tended to center on estimating the cost of the illness [26–29].

Therefore, the objective of the present study was to analyze the association between obesity and health system utilization (considering services related to hypertension and/or diabetes), based on representative data for the Brazilian population.

## Methods

### Study design and data source

A cross-sectional study of secondary data from the National Health Survey (PNS) was conducted. The PNS is a population-based household survey in Brazil, by the Ministry of Health in partnership with the Brazilian Institute of Geography and Statistics (IBGE) [30]. The PNS sought to assess the performance of Brazil’s national health system, the health status of the Brazilian population, and surveillance of chronic non-communicable diseases and associated risk factors [30]. The data were collected between August 2013 and February 2014 by the IBGE [2].

### Study population and sampling

The National Health Survey assessed a representative sample of the Brazilian population aged ≥18 years, comprising dwellers of private households situated nationwide. Three-stage cluster sampling was employed. The first stage involved census sectors; the second, households; and the third, dwellers aged ≥18 years residing in randomly selected households. The selection at each stage was done using simple random sampling [30]. A total of 64,348 household interviews and 60,202 individual interviews were carried out [30–32].

### Data collection

A three-part questionnaire was applied to collect the PNS data: 1) household; 2) household dwellers; and 3) individual. The individual questionnaire relevant to the study was answered by only one dweller, aged at least 18 years, randomly selected by the interviewer, using a hand-held personal computer (PDA - Personal Digital Assistant). The same device was used to store the other information collected [30].

Weight and height were measured using a digital scale and portable stadiometer, respectively. The final weight and height values were the arithmetic mean of two measurements. All collection agents were trained to carry out the interviews using the PDA and were duly qualified to take all anthropometric measurements. A detailed description of the data collection procedure is available elsewhere in a specific publication [30].

### Study variables

#### Exposure

The main exposure analyzed was the body mass index (BMI – weight/height^2^) category of the individuals. BMI was categorized as follows: underweight (BMI < 18.5 Kg/m^2^) or normal weight (BMI 18.5–24.9 Kg/m^2^), hereafter referred to as under/normal weight (considering BMI < 25.0 Kg/m^2^); overweight (BMI = 25.0–29.9 Kg/m^2^); and obesity (BMI ≥ 30.0 Kg/m^2^), according to the WHO [33]. Due to the small number of individuals in the underweight category (2.5%), these were considered together with normal weight individuals. The sensitivity analysis performed (data not shown) confirmed this possibility with no harm to the estimative.

All subjects that answered the individual part of the questionnaire and had height and weight data measured available were eligible for the analyses. Women who stated they were pregnant were excluded.

#### Outcomes

The outcomes analyzed were those related to the use of health services for systemic arterial hypertension and diabetes mellitus, NCDs commonly associated with obesity, and high levels of morbi-mortality [10, 11, 29, 34, 35]. For these diseases, the PNS collected information on a range of health services, allowing investigations of all levels of healthcare (primary, secondary, tertiary) [36]. We assessed both diseases individually and simultaneously, considering their existence at the same time among the individuals.

The information on health service utilization was provided by individuals who reported having clinically diagnosed hypertension and/or diabetes. These individuals were probed on the use of the following health services: 1) routine visits to the doctor or health service for the disease; 2) have done all exams prescribed by the doctor; 3) referrals to a specialist (including consultations); and 4) hospital admission for the disease or related complication [36]. In the case of individuals with both diseases, we considered the utilization of the health service due to at least one of them.

For the question on routine visits to the doctor or health service for hypertension and/or diabetes, respondents who reported visiting the doctor or medical services sporadically, i.e. only in the occurrence of problems, were not counted as positive cases. With regard to the question on referrals to a specialist, besides a “Yes” answer, the option “There was no referral because the consultation was conducted directly with the specialist” was also considered a positive case. The following medical specialties were surveyed by the PNS: cardiology or nephrology, for individuals diagnosed with hypertension; and cardiology, nephrology, endocrinology, or ophthalmology, for those diagnosed with diabetes [36].

The four outcomes were expressed as binary variables, representing the use or non-use of the above-cited services.

#### Covariables

The sociodemographic variable used to describe the sample and adjust the analyses were: educational level (no formal education or primary incomplete, primary complete or secondary incomplete, secondary complete or higher incomplete, higher complete or greater), age (stratified into 18–29 / 30–39 / 40–49 / 50–59 / ≥60 years), race/skin color (white, black, mixed-race, yellow, indigenous), macroregion (North, Northeast, Mid-West, Southeast, South) and residence area (urban, rural).

### Data analysis

Descriptive statistical analysis of all variables was performed to determine the distribution of the characteristics of the study population. Relative frequencies were calculated to analyze the relationship between BMI categories, sociodemographic characteristics, and the report of health service use for hypertension and/or diabetes. All analyses were stratified by sex (male/female), as opposed to using this parameter merely for adjusting, thereby enabling observation of the relationship of this variable with possible explanations of associations [37, 38].

The associations between the three BMI categories and health service use were analyzed using Poisson regression models (crude, and adjusted for education, age, race/skin color, macroregion, and residence area, considering results of previous studies [39, 40], with the calculation of prevalence ratios (PR) and their respective 95% confidence intervals (95%CI). Further exploratory analyses were also carried out to assess the association of the different degrees of obesity with the outcomes. Differences were deemed statistically significant when there was no overlapping of confidence intervals.

All data analyses were carried out using the statistics package Stata version 12.1 (Stata Corp., College Station, USA), with the *survey* module, that considers the effects of complex sampling of the PNS, enabling extrapolation of the results for the Brazilian population as a whole.

### Ethics aspects

We declare that PNS and our analyses were carried out in accordance with the relevant national and international guidelines and regulations. The National Health Survey was approved by the National Commission on Research Ethics for Humans (Conep) of the National Board of Health (CNS), under permit n° 328.159. All study participants signed the Free and Informed Consent Form. We used data from the PNS, collected by the IBGE, and available online (https://www.ibge.gov.br/estatisticas/sociais/saude/9160-pesquisa-nacional-de-saude.html?=&t=microdados). The information contained in the database is confidential since specific data about each household such as identification of the household members, address and telephone are excluded.

## Results

The 2013 PNS interviewed 60,202 individuals aged ≥18 years. Of the original total, 800 individuals (1.3%) were excluded for missing weight and/or height data, giving a total sample of 59,402 participants for analysis. This population had a mean weight of 70.4 Kg (± 15.2 Kg), height of 1.63 m (± 9.7 cm), and mean BMI of 26.5 Kg/m^2^ (data not shown). Of the population assessed, 36.4% had overweight, and over a fifth (20.8%), obesity (data not shown).

The percentage distribution for the three BMI categories, according to sociodemographic characteristics and sex, is summarized in Table 1. Nationally, 16.8% of men had obesity, versus 24.3% of women.

**Table 1:** Distribution of population by BMI, sociodemographic characteristics, and sex (*n* = 59,402). PNS, Brazil, 2013

Sociodemographic characteristics	Male (***n*** = 25,920)						Female (***n*** = 33,482)					
	BMI Category^b^						BMI Category^b^					
	Under/Normal weight^a^	95%CI	Overweight	95%CI	Obesity	95%CI	Under/Normal weight^a^	95%CI	Overweight	95%CI	Obesity	95%CI
**BRAZIL**	44.5	43.4–45.6	38.8	37.7–39.8	16.8	15.9–17.6	42.0	41.0–43.0	33.7	32.8–34.5	24.3	23.5–25.1
**Macroregion**												
North	45.8	43.4–48.2	39.3	36.9–41.7	14.9	13.2–16.6	46.8	44.9–48.7	33.3	31.5–35.1	19.8	18.3–21.4
Northeast	49.2	47.3–51.0	36.8	35.1–38.5	14.0	12.8–15.3	44.5	42.9–46.1	34.1	32.6–35.5	21.4	20.1–22.7
Midwest	42.9	40.7–45.1	38.3	35.8–40.7	18.8	17.2–20.5	43.3	41.5–45.0	32.0	30.3–33.8	24.7	23.1–26.3
Southeast	43.3	41.2–45.3	39.4	37.5–41.2	17.4	15.8–19.0	40.2	38.5–41.9	33.7	32.1–35.2	26.1	24.6–27.6
South	39.7	37.5–42.0	40.4	37.8–43.0	19.9	17.9–21.9	39.8	37.3–42.4	33.8	31.8–35.9	26.3	24.2–28.5
**Residence area**												
Urban	42.6	41.3–43.8	39.6	38.5–40.8	17.8	16.8–18.8	41.7	40.6–42.8	33.6	32.6–34.5	24.7	23.8–25.6
Rural	55.3	53.0–57.6	33.7	31.8–35.6	11.0	9.4–12.5	44.2	41.7–46.6	34.1	32.0–36.3	21.7	19.8–23.6
**Age (years)**												
18–29	60.0	58.0–62.0	29.4	27.5–31.3	10.6	9.1–12.0	62.5	60.6–64.5	23.4	21.7–25.0	14.1	12.6–15.6
30–39	40.9	38.6–43.2	42.0	39.8–44.3	17.1	15.6–18.5	42.4	40.6–44.3	33.9	32.0–35.7	23.7	22.0–25.4
40–49	36.0	33.8–38.2	43.2	41.0–45.4	20.8	18.8–22.8	33.3	31.2–35.4	38.6	36.6–40.6	28.1	26.2–30.0
50–59	34.6	31.8–37.3	44.2	41.3–47.0	21.3	18.9–23.6	29.4	27.4–31.4	37.8	35.6–40.1	32.7	30.3–35.1
≥ 60	42.3	39.9–44.7	39.7	37.2–42.3	17.9	15.7–20.2	34.0	32.0–36.1	38.4	36.4–40.4	27.6	25.7–29.4
**Race/skin color**												
White	40.2	38.6–41.8	40.6	39.0–42.2	19.1	17.8–20.5	41.8	40.4–43.2	33.3	32.0–34.6	24.9	23.7–26.0
Black	46.8	43.3–50.2	35.7	32.4–38.9	17.6	14.6–20.5	41.2	38.1–44.2	30.5	27.8–33.3	28.3	25.6–31.0
Yellow	57.6	47.4–67.9	35.3	25.4–45.2	7.0	2.8–11.2	53.5	43.6–63.4	31.4	22.6–40.1	15.1	9.29–20.9
Mixed-race	48.3	46.7–49.9	37.4	36.0–38.9	14.2	13.1–15.3	42.2	40.8–43.5	34.8	33.4–36.1	23.0	21.9–24.2
Indigenous	47.8	35.4–60.3	36.9	24.9–48.8	15.3	6.6–24.0	40.8	30.2–51.4	35.4	25.0–45.8	23.8	14.7–32.9
**Education**												
No formal schooling or incomplete primary	47.8	46.1–49.5	37.1	35.5–38.7	15.0	13.9–16.2	35.0	33.6–36.4	36.4	34.9–37.8	28.6	27.3–30.0
Complete primary or incomplete secondary	50.8	48.1–53.5	33.5	31.1–36.0	15.7	13.5–17.9	42.1	39.6–44.7	33.8	31.3–36.2	24.1	22.0–26.1
Complete secondary or incomplete higher	42.0	40.1–43.9	40.4	38.5–42.2	17.6	16.1–19.1	47.0	45.4–48.6	31.3	29.8–32.8	21.7	20.3–23.1
Complete higher or above	30.6	27.8–33.4	47.3	44.3–50.4	22.1	19.5–24.7	49.2	46.5–51.8	31.8	29.5–34.0	19.1	17.0–21.1

^a^Includes underweight individuals

^b^Under/Normal weight: BMI < 25.0 Kg/m^2^ / Overweight: BMI = 25.0–29.9 Kg/m^2^ / Obesity: BMI ≥ 30.0 Kg/m^2^

For both sexes, the Southern region of the country accounted for the greatest number of individuals with obesity and dwellers in urban areas. The obesity rate increased with age, particularly from 30 years and older, where the prevalence of obesity was double among those aged 50–59 years, compared the 18–29 years age group. Regarding rates among older adults, 27.6% of women and 17.9% of men were with obesity. Men with obesity had a greater probability of being white, and progressively higher obesity rates were observed with increasing education level. For women, obesity was more frequent in black individuals and those with no formal schooling or with incomplete primary (Table 1).

The prevalence of individuals diagnosed with hypertension and/or diabetes in the 59.402 participants surveyed, together with rates of health service utilization for the two diseases, are given in Table 2. Overall, 21.8% had hypertension, 6.8% had diabetes and 3.9% had both diseases (data not shown). Of the hypertension group, 38.1% were with overweight and 36.3% with obesity, whereas in the diabetes group, 38.2% were with overweight and 37.0% with obesity (data not shown).

**Table 2:** Type of health service used by disease and sex (*n* = 59,402). PNS, Brazil, 2013

Type of health service used due to the diseases	Disease Diagnosed	Male		Female	
		%	95%CI	%	95%CI
**Prevalence of disease diagnosed**	SAH	**18.7**	17.9–19.4	**24.7**	24.0–25.5
	DM	**5.9**	5.4–6.5	**7.5**	7.0–8.0
	SAH and DM	**3.1**	2.7–3.5	**4.7**	4.3–5.1
**Routine visits to doctor or health service**	SAH	**9.9**	9.3–10.5	**15.5**	14.8–16.2
	DM	**3.4**	3.0–3.8	**5.0**	4.6–5.4
	SAH and DM	**3.7**	3.3–4.2	**5.7**	5.3–6.2
**Prescribed exams done**	SAH	**12.4**	11.8–13.1	**17.4**	16.7–18.0
	DM	**4.3**	3.8–4.7	**5.4**	5.0–5.8
	SAH and DM	**4.6**	4.1–5.0	**6.0**	5.6–6.4
**Referral to specialist**^a^	SAH	**9.1**	8.5–9.7	**12.0**	11.3–12.6
	DM	**2.6**	2.3–3.0	**3.2**	2.8–3.5
	SAH and DM	**3.2**	2.8–3.6	**4.0**	3.7–4.4
**Hospitalization for disease or related complication**	SAH	**2.3**	2.0–2.6	**3.7**	3.3–4.0
	DM	**0.8**	0.6–1.0	**0.9**	0.7–1.0
	SAH and DM	**1.1**	0.9–1.3	**1.8**	1.6–2.1

*95%IC* 95% Confidence Interval, *SAH* Systemic Arterial Hypertension, *DM* Diabetes Mellitus

^a^Includes previous consultation

Rates of the two diseases were higher in women, as were figures for all outcomes investigated, particularly for the variables “routine visits to doctor or health service for hypertension” (15.5% versus 9.9% in men) and “exams done due hypertension” (17.4% versus 12.4% in men) (Table 2).

Regarding the relationship between BMI category and health service utilization, a gradient was observed, with commensurately higher rates of use of all services among subjects with overweight and obesity, compared with under/normal weight individuals. This pattern was evident for both sexes but was stronger among women. Overall, 8.3% (95%CI 7.5–9.1) of women of under/normal weight reported routine visits to the doctor or health service for hypertension, compared to 25.1% (95%CI 23.6–26.7) of women with obesity. Hospital admission for diabetes was the only outcome that showed no statistically significant difference for BMI category, in both sexes (Table 3).

**Table 3:** Type of health service used by disease, sex and BMI category (*n* = 59,402). PNS, Brazil, 2013

Type of health service used due to the diseases	Disease Diagnosed	Male						Female					
		BMI Category^a^						BMI Category^a^					
		Under/Normal weight^b^		Overweight		Obesity		Under/Normal weight^b^		Overweight		Obesity	
		%	95%CI	%	95%CI	%	95%CI	%	95%CI	%	95%CI	%	95%CI
**Routine visits to doctor or health service**	**SAH**	**5.9**	5.2–6.6	**10.7**	10.0–11.8	**18.6**	16.5–20.7	**8.3**	7.5–9.1	**16.8**	15.6–18.1	**25.1**	23.6–26.7
	**DM**	**2.2**	1.7–2.6	**3.6**	2.8–4.3	**6.6**	5.3–7.8	**2.5**	2.1–2.9	**5.7**	4.9–6.4	**8.1**	7.1–9.0
	**SAH and DM**	**2.2**	1.8–2.8	**3.9**	3.2–4.8	**7.2**	6.0–8.6	**2.7**	2.3–3.2	**6.3**	5.5–7.1	**9.7**	8.6–10.8
**Exams done**	**SAH**	**7.8**	7.0–8.6	**12.8**	11.7–13.9	**23.7**	21.5–25.9	**9.4**	8.6–10.3	**19.1**	17.8–20.4	**27.6**	26.0–29.3
	**DM**	**2.8**	2.2–3.3	**4.4**	3.6–5.2	**8.0**	6.6–9.3	**2.8**	2.3–3.2	**6.1**	5.3–6.8	**8.6**	7.6–9.6
	**SAH and DM**	**2.9**	2.4–3.5	**4.6**	3.9–5.5	**8.7**	7.4–10.2	**3.0**	2.6–3.5	**6.8**	6.0–7.6	**9.6**	8.6–10.7
**Referral to specialist**^c^	**SAH**	**5.4**	4.7–6.1	**9.8**	8.8–10.9	**17.3**	15.3–19.3	**6.4**	5.6–7.2	**13.7**	12.6–14.8	**18.4**	17.1–19.8
	**DM**	**1.7**	1.2–2.1	**2.8**	2.2–3.4	**4.9**	3.8–6.0	**1.4**	1.1–1.7	**3.6**	3.0–4.2	**5.4**	4.6–6.2
	**SAH and DM**	**2.0**	1.6–2.6	**3.2**	2.6–4.0	**6.0**	4.9–7.3	**1.7**	1.3–2.0	**4.7**	4.1–5.5	**6.9**	6.1–7.8
**Hospitalization**	**SAH**	**1.4**	1.1–1.8	**2.3**	1.7–2.8	**4.6**	3.4–5.8	**1.9**	1.5–2.2	**3.8**	3.2–4.4	**6.4**	5.5–7.2
	**DM**	**0.7**	0.4–0.9	**0.7**	0.4–1.0	**1.4**	0.8–2.0	**0.6**	0.4–0.7	**0.9**	0.6–1.2	**1.4**	0.9–1.8
	**SAH and DM**	**0.8**	0.5–1.1	**0.9**	0.7–1.3	**2.3**	1.6–3.2	**0.8**	0.6–1.0	**2.0**	1.6–2.5	**3.2**	2.5–4.0

*95%CI* 95% Confidence Interval, *SAH* Systemic Arterial Hypertension, *DM* Diabetes Mellitus

^a^Under/Normal weight: BMI < 25.0 Kg/m^2^ / Overweight: BMI = 25.0–29.9 Kg/m^2^ / Obesity: BMI ≥ 30.0 Kg/m^2^

^b^Includes underweight individuals. ^c^Includes previous consultation

The results of the multiple regression model revealed that higher prevalence ratios were associated with individuals with overweight and obesity, where this also held true for all outcomes measured and for both sexes. After adjusting for sociodemographic variables, the strength of association was attenuated for all outcomes. However, adjusted Prevalence Ratios (aPRs) still showed around double (or greater) the rates of health service use among subjects with obesity versus under/normal weight individuals (Table 4).

**Table 4:** Association of obesity and type of service by sex and disease diagnosed (*n* = 59,402). PNS, Brazil, 2013

Type of health service used	Disease Diagnosed	Crude Model								Adjusted Model^a^							
		Male				Female				Male				Female			
		Overweight^b^		Obesity^b^		Overweight^b^		Obesity^b^		Overweight^b^		Obesity^b^		Overweight^b^		Obesity^b^	
		PR^c^	95%CI	PR^c^	95%CI	PR^c^	95%CI	PR^c^	95%CI	aPR^c^	95%CI	aPR^c^	95%CI	aPR^c^	95%CI	aPR^c^	95%CI
**Routine visits to doctor or health service**	SAH	**1.79**	1.52–2.11	**3.12**	2.65–3.68	**2.01**	1.78–2.28	**3.00**	2.66–3.38	**1.44**	1.23–1.68	**2.34**	1.99–2.74	**1.45**	1.29–1.63	**2.03**	1.81–2.26
	DM	**1.65**	1.22–2.22	**3.04**	2.26–4.09	**2.25**	1.82–2.78	**3.20**	2.62–3.90	**1.30**	0.98–1.72	**2.19**	1.64–2.93	**1.62**	1.32–1.99	**2.14**	1.75–2.61
	SAH and DM	**1.75**	1.33–2.30	**3.20**	2.41–4.27	**2.33**	1.90–2.86	**3.59**	2.98–4.32	**1.38**	1.06–1.79	**2.32**	1.75–3.07	**1.66**	1.36–2.03	**2.36**	1.96–2.84
**Exams done**	SAH	**1.63**	1.42–1.88	**3.02**	2.63–3.48	**2.01**	1.79–2.25	**2.90**	2.59–3.24	**1.32**	1.16–1.50	**2.28**	1.99–2.60	**1.47**	1.32–1.63	**2.02**	1.82–2.24
	DM	**1.61**	1.24–2.08	**2.90**	2.22–3.79	**2.16**	1.77–2.62	**3.06**	2.53–3.69	**1.27**	0.99–1.62	**2.10**	1.62–2.72	**1.56**	1.28–1.89	**2.06**	1.70–2.48
	SAH and DM	**1.59**	1.25–2.04	**2.99**	2.32–3.86	**2.23**	1.84–2.70	**3.14**	2.63–3.76	**1.26**	1.00–1.59	**2.17**	1.70–2.78	**1.59**	1.32–1.91	**2.09**	1.75–2.49
**Referral to specialist**^d^	SAH	**1.82**	1.53–2.16	**3.19**	2.68–3.79	**2.12**	1.84–2.46	**2.85**	2.47–3.30	**1.43**	1.22–1.67	**2.33**	1.98–2.75	**1.55**	1.35–1.77	**1.95**	1.70–2.23
	DM	**1.66**	1.18–2.35	**2.92**	2.07–4.11	**2.61**	1.96–3.47	**3.87**	2.94–5.09	**1.24**	0.89–1.73	**2.01**	1.44–2.81	**1.86**	1.40–2.48	**2.56**	1.94–3.38
	SAH and DM	**1.58**	1.19–2.09	**2.91**	2.14–3.94	**2.85**	2.21–3.67	**4.14**	3.24–5.30	**1.19**	0.90–1.56	**2.01**	1.49–2.71	**2.02**	1.57–2.59	**2.70**	2.11–3.46
**Hospitalization**	SAH	**1.60**	1.13–2.26	**3.21**	2.31–4.45	**2.02**	1.56–2.60	**3.37**	2.65–4.28	**1.35**	0.96–1.88	**2.55**	1.81–3.61	**1.48**	1.15–1.89	**2.34**	1.85–2.95
	DM	**1.04**	0.60–1.81	**2.11**	1.17–3.80	**1.60**	0.99–2.57	**2.42**	1.52–3.85	**0.95**	0.57–1.60	**1.81**	1.01–3.25	**1.17**	0.73–1.87	**1.66**	1.04–2.63
	SAH and DM	**1.24**	0.76–2.03	**2.99**	1.83–4.89	**2.52**	1.79–3.56	**4.04**	2.90–5.63	**1.10**	0.70–1.75	**2.48**	1.54–3.99	**1.80**	1.28–2.52	**2.66**	1.92–3.69

*PR* Prevalence Ratio, *aPR* Adjusted Prevalence Ratios, *95%CI* 95% Confidence Interval, *SAH* Systemic Arterial Hypertension, *DM* Diabetes Mellitus

^a^Model adjusted for macroregion, residence area, age, race/skin color and education

^b^Overweight: BMI = 25.0–29.9 Kg/m^2^ / Obesity: BMI ≥ 30.0 Kg/m^2^

^c^Reference category = Underweight + Normal weight. ^d^Includes previous consultation

Comparing the health services assessed, on adjusted models, men with obesity had higher PR (Prevalence Ratios), especially regarding health service use for hypertension, particularly hospital admission (aPR = 2.55; 95%CI 1.81–3.61). In women with obesity, higher PR were found regarding health service use for diabetes, particularly referrals or consultations with specialists (aPR = 2.56; 95%CI 1.94–3.38) (Table 4).

Individuals diagnosed with both diseases tended to present an increased risk for using practically all types of services investigated. Higher PR was observed among women with obesity, mainly in relation to being referred to a specialist (aPR = 2.70; 95%CI 2.11–3.46) and hospitalization (aPR = 2.66; 95%CI 1.92–3.69) (Table 4).

Further analyses were carried out using BMI stratified into six categories, namely: underweight, normal weight, overweight, obesity grade I, obesity grade II and obesity grade III. Given there was no statistically significant difference for obesity grade, despite the pattern of greater service use with increasing BMI, the pooled form of the variable was retained. This lack of difference is likely explained by the low proportion of individuals with higher grades of obesity contained in the sample. These results can be found in the supplementary material.

## Discussion

The present study found greater health service utilization by individuals with overweight and obesity, of both sexes, compared to under/normal-weight individuals. Individuals with hypertension or diabetes may need to use health services to a lesser or greater degree, and overweight and obesity influenced the extent of this utilization. The presence of obesity doubled the utilization of the health services investigated. One explanation for this higher use is that obesity may worsen the clinical condition of individuals with hypertension and/or diabetes, leading to the need for greater, or more frequent, health care. Another hypothesis is that, since obesity is a risk factor for other diseases, individuals with obesity needed to use health services more often after developing other NCDs, such as hypertension and diabetes.

These findings corroborate results seen in some high-income countries, such as Ireland [41], the USA (United States of America) [42], and Canada [43]. In Ireland, a representative study of the middle-aged and older adult population found that all obesity categories were associated with a higher number of GP visits [41]. In the USA, an eight-year retrospective cohort study of young and middle-aged adults revealed that obesity was associated with a higher rate of outpatient consultations, ER visits, and hospitalizations. Individuals with obesity had a two-fold greater risk of visiting the emergency room than normal-weight subjects, and weight gain over time was also associated with a higher risk of emergency room use [42]. In a five-year study, Canadians with a BMI ≥ 35 Kg/m^2^ made more GP visits than their normal-weight counterparts. Results of the study highlighted the burden of obesity, particularly in primary health care [43].

The present study identified an association between obesity and greater health service utilization in Brazil, a middle-income country. In addition, this increased use by individuals with obesity was found across all three levels of healthcare.

Greater health service utilization raises health costs, both in the private and public systems, particularly for higher-cost services such as hospital admissions, exams, and consultations with specialists [26, 28, 41, 42, 44]. Moreover, high demand for less accessible services, such as those requiring specialized staff and those that have limited hospital beds, can overload the health system, precluding care delivery or reducing the duration or quality of the service provided, since there will be much demand for the same service [45].

The study results showed higher health service utilization by women. The literature shows, among other factors, that culturally women have a greater tendency to seek healthcare services, compared to men [17, 24, 26, 46, 47].

Besides the fact that caring for one’s health is not regarded as a male pursuit, given that most primary and secondary healthcare services are not available during night-time hours or weekends, men, who still make up the majority of the formal workforce [48], may experience more difficulty accessing these services [24, 47]. Although not explained solely by access, it remains a key determinant of health service utilization [15]. Furthermore, the shortcomings of public services with regard to care, where users often face long waiting times and do not always have their health needs resolved in a single session, may pose another barrier to utilization by men. On top of these factors, men more often display a mindset of somehow “being unsusceptible to illness” [24, 47], reflecting a poorer perception of their need for healthcare, which itself may stem from lower use of these services [16].

Women also had the highest rates of hypertension and diabetes. This finding may suggest that, due to their greater propensity to seek healthcare services, women undergo more diagnostic exams and hence have a clearer picture of their true health situation [17]. Women also made greater use of services for follow-up treatment of the diseases (routine visits to doctor or health services use to realize exams). More rigorous disease follow-up suggests greater control and prevention of complications related to the disease [34].

The analysis of health service utilization for hypertension revealed a higher risk among the group of men with obesity for regular visits to doctor and for exams, compared to the utilization of these same services by women with obesity. In addition to obesity being associated with an increased risk of using all health services investigated, these findings reveal that gender also influenced the use of two of the four types of services investigated. In general, the risk of complications related to arterial hypertension was higher among men [49], where presenting obesity is expected to worsen the clinical condition of these individuals. This scenario, together with less frequent follow-up (and consequently less control) of the disease, would normally increase the need for the use of these services.

The cross-sectional nature of the present study does not allow inferring whether obesity preceded the diseases (given obesity is a risk factor for them), or whether weight gain (and consequently increased BMI) occurred after diagnosis of the diseases investigated. Nevertheless, in the present study, the association between the occurrence of obesity and greater health service utilization was clear.

This study has some limitations. It was not possible to distinguish between the two types of diabetes (types 1 and 2). Given that type 2 diabetes is more strongly linked with lifestyle, this type can better explain an association with obesity. However, because type 2 diabetes is more prevalent than type 1 [50], most individuals with this sub-diagnosis probably had the type 2.

Regarding race/skin color, two categories are composed of small numbers of individuals (indigenous and yellow), so it is difficult to conclude about them due to low precision. However, we chose to keep with these categories because we understand that is relevant to present descriptive data on this population, recognize their existence, and reflect the need for specific surveys related to these groups.

About the question: “routine visits to the doctor or health service for the disease”, it was not possible to quantify the number of visits, rendering the variable subject to interpretation, i.e. the concept of “routine” could have been interpreted differently by respondents.

In addition, this was a household-based study in which data on the presence of diseases and use of health services were reported by patients as opposed to being drawn from medical records, where this self-reporting may have led to under or overestimations. However, there is growing the use of self-reported information on morbidity in regular health surveys, despite its limitations, owing to the faster data collection and publication afforded. These factors, together with the inherently lower costs, make this approach useful and timely for health surveillance actions [35]. Also, the PNS did not collect data on health service access and we focused only on services related to hypertension and diabetes since only for those would be possible to assess all levels of healthcare (primary, secondary, tertiary).

It is also important to mention that, despite the data from the PNS-2019 being available, the survey had measured data only for a small subsample and self-referred data for all individuals. The present work analyzed the data from the PNS-2013 because, in this survey, the weight and height data were measured by trained professionals for the whole population, which eliminates reporting bias. As the objective of the present study was to analyze the association between obesity and the use of health services, and not to describe the prevalence, it is believed that the magnitude of the association is not influenced by this temporal difference.

Lastly, previous studies suggest that increased health service utilization by individuals with overweight or obesity is only partly explained by chronic health conditions [51, 52]. Thus, the findings of the present study may be underestimated, as the use of health services directly related to obesity was not investigated.

The underestimation of health services use by individuals with obesity may also be associated with social stigma in this group. A systematic review of observational studies on this issue found that 19.2–41.8% of individuals with obesity had been subject to discrimination [53], including healthcare-related discrimination, which can make them use less health services than they really need.

Therefore, the present findings likely represent a lower rate of use of these services than actually required by individuals with obesity. In addition, only two NCDs associated with obesity were assessed, while the literature shows obesity to be associated with many other NCDs [4, 7, 9, 54]. In addition, although in the present study no significant difference was found in the utilization of health services between individuals with an isolated or simultaneous diagnosis of hypertension and diabetes, it is known that the presence of multimorbidity may interfere with this utilization [19], which is a relevant point to be considered, especially for studies that assess a greater number of obesity-related diseases.

Taken together, the results showed that individuals with obesity made greater use of health services than their under/normal-weight counterparts, for both low and high-complexity services. Results of population-based studies, such as the present investigation, can significantly contribute to the planning, formulation, and management of health policies, particularly in the public sphere. Future studies on this subject should include data on access to health services and investigate health service utilization associated with the direct and indirect costs of obesity.

## Conclusion

Brazilian individuals with obesity (both male and female) made roughly double the use of all health care services investigated: routine visits to a general doctor; referrals/consultations with a specialist; prescribed exams done; and hospital admission (considering services related to hypertension and/or diabetes), compared with under/normal-weight individuals. Therefore, besides other consequences, the presence of obesity demands greater human resources and materials to the national health system.

The present study, based on population representative data, identified an association between obesity and greater health service utilization in a middle-income country, finding results across all three levels of healthcare. These findings aim to contribute to national and international public health.

## Supplementary Information

**Additional file 1 **Health service use according to type, BMI and sex (*n* = 59,402). PNS, Brazil, 2013.

## Data Availability

The data that support the findings of this study are openly available in Brazilian Institute of Geography and Statistics (IBGE) at https://www.ibge.gov.br/estatisticas/sociais/saude/9160-pesquisa-nacional-de-saude.html?=&t=microdados.

## Abbreviations

aPR: adjusted Prevalence Ratio
BMI: Body Mass Index
CNS: National Board of Health
Conep: National Commission on Research Ethics for Humans
DM: Diabetes Mellitus
IBGE: Brazilian Institute of Geography and Statistics
NCD: Non-Communicable Disease
PDA: Personal Digital Assistant
PNS: National Health Survey
PR: Prevalence Ratio
SAH: Systemic Arterial Hypertension
USA: United States of America
WHO: World Health Organization
95%CI: 95% Confidence Interval
