# Supplementary material for: Obesity and health service utilization in Brazil: data from the National Health Survey
Source: BMC Public Health. 2022 Aug 2;22:1474. doi: 10.1186/s12889-022-13906-2 (PMC9344684; doi:10.1186/s12889-022-13906-2)
Supplement: Supplementary file 1 — Additional file 1 Health service use according to type, BMI and sex (n = 59,402). PNS, Brazil, 2013. [file 12889_2022_13906_MOESM1_ESM.pdf]

Supplementary material. Health service use according to type, BMI and sex (n = 59,402). PNS, Brazil, 2013.

Type of health service used by sex	BMI Categories[1]											
	Underweight		Normal weight		Overweight		Obesity I		Obesity II		Obesity III	
	%	95%CI	%	95%CI	%	95%CI	%	95%CI	%	95%CI	%	95%CI
**Routine visits to doctor or health service for SAH**	**5.3**	3.4-7.1	**7.3**	6.7-7.9	**13.7**	12.9-14.6	**20.1**	18.7-21.6	**28.3**	25.2-31.4	**30.6**	25.5-35.7
Male	**3.1**	1.0-5.1	**6.1**	5.4-6.8	**10.7**	10.0-11.8	**17.0**	14.7-19.4	**22.0**	16.5-27.6	**29.2**	18.8-39.6
Female	**6.7**	4.0-9.4	**8.4**	7.5-9.3	**16.8**	15.6-18.1	**22.3**	20.5-24.0	**31.6**	28.1-35.2	**31.0**	25.2-36.9
**Routine visits to doctor or health service for DM**	**2.4**	0.9-3.8	**2.3**	2.0-2.6	**4.6**	4.1-5.1	**6.9**	6.0-7.7	**8.3**	6.6-9.9	**11.1**	7.5-14.7
Male	**2.9**	0.0-5.9	**2.1**	1.6-2.6	**3.6**	2.8-4.3	**6.2**	4.9-7.6	**6.8**	3.6-10.0	**10.4**	2.3-18.4
Female	**2.0**	0.7-3.3	**2.5**	2.1-3.0	**5.7**	4.9-6.4	**7.3**	6.1-8.4	**9.0**	7.1-11.0	**11.3**	7.4-15.3
**Routine visits to doctor or health service (individuals with SAH and DM)***	**2.4**	1.0-3.8	**2.5**	2.2-2.8	**5.1**	4.5-5.6	**7.8**	6.9-8.7	**10.3**	8.2-12.4	**12.9**	9.2-16.6
Male	**2.9**	-0.05-5.9	**2.2**	1.7-2.7	**3.9**	3.2-4.7	**6.8**	5.3-8.2	**7.8**	4.5-11.1	**11.8**	3.7-19.8
Female	**2.0**	0.7-3.3	**2.7**	2.3-3.2	**6.3**	5.5-7.1	**8.5**	7.3-9.8	**11.7**	9.0-14.4	**13.2**	9.1-17.4
**Exams done for SAH**	**6.0**	4.1-7.9	**8.8**	8.2-9.5	**15.9**	15.1-16.8	**24.0**	22.5-25.6	**29.2**	26.0-32.4	**37.1**	32.0-42.3
Male	**3.4**	1.4-5.4	**8.1**	7.2-8.9	**12.8**	11.7-13.9	**21.9**	19.5-24.3	**27.8**	21.5-34.1	**36.6**	25.8-47.5
Female	**7.7**	4.9-10.6	**9.6**	8.7-10.4	**19.1**	17.8-20.4	**25.5**	23.5-27.5	**30.0**	26.5-33.4	**37.3**	31.4-43.2
**Exams done for DM**	**2.5**	1.1-3.9	**2.8**	2.4-3.1	**5.2**	4.7-5.8	**8.0**	7.1-9.0	**8.6**	6.7-10.5	**11.2**	7.8-14.7
Male	**3.3**	0.3-6.3	**2.7**	2.2-3.3	**4.4**	3.6-5.2	**7.8**	6.3-9.3	**7.9**	4.7-11.1	**11.5**	3.4-19.6
Female	**2.0**	0.7-3.2	**2.8**	2.4-3.3	**6.1**	5.3-6.8	**8.2**	7.0-9.4	**8.9**	6.8-11.0	**11.2**	7.4-15.0
**Exams done (individuals with SAH and DM)***	**2.8**	1.4-4.3	**3.0**	**2.6-3.3**	**5.7**	**5.1-6.3**	**8.7**	**7.7-9.6**	**9.8**	**7.8-11.9**	**12.9**	**9.3-16.6**
Male	**3.4**	0.4-6.4	**2.9**	2.3-3.4	**4.6**	3.8-5.5	**8.4**	6.9-9.9	**9.0**	5.7-12.4	**12.4**	4.3-20.5
Female	**2.4**	1.0-3.8	**3.1**	2.6-3.6	**6.8**	6.0-7.8	**8.8**	7.6-10.1	**10.3**	7.9-12.6	**13.1**	9.1-17.1
**Referral to specialist for SAH (includes consultation)**	**2.4**	0.9-4.0	**2.7**	2.3-3.0	**5.4**	4.8-6.0	**7.1**	6.2-8.0	**8.3**	6.7-9.9	**10.9**	7.6-14.2
Male	**1.4**	-0.2-3.1	**2.6**	2.1-3.0	**4.6**	3.7-5.4	**7.0**	5.4-8.6	**6.8**	4.1-9.5	**14.6**	5.7-23.6
Female	**3.1**	0.8-5.4	**2.8**	2.3-3.3	**6.2**	5.3-7.0	**7.2**	6.2-8.3	**9.0**	7.1-11.0	**9.8**	6.4-13.1
**Referral to specialist for DM (includes consultation)**	**1.0**	-1.6-2.2	**0.7**	0.5-0.8	**1.6**	1.2-1.9	**1.8**	1.3-2.2	**2.7**	1.7-3.7	**3.4**	1.7-5.2
Male	**1.9**	-0.9-4.8	**0.6**	0.4-0.9	**1.5**	1.0-2.0	**1.6**	0.9-2.2	**3.3**	1.1-5.5	**1.4**	-0.4-3.4
Female	**0.4**	0.0-0.9	**0.7**	0.4-1.0	**1.6**	1.2-2.1	**1.9**	1.3-2.5	**2.4**	1.4-3.4	**4.1**	1.8-6.3
**Referral to specialist (includes consultation) (individuals with SAH and DM)***	**1.8**	0.5-3.1	**1.8**	1.6-2.1	**4.0**	3.5-4.5	**6.1**	5.3-7.0	**7.2**	5.7-8.8	**8.3**	5.9-10.8
Male	**2.8**	-0.2-5.8	**2.0**	1.6-2.5	**3.2**	2.6-3.9	**5.8**	4.4-7.1	**6.8**	3.9-9.6	**5.8**	2.0-9.5
Female	**1.1**	0.3-2.0	**1.7**	1.3-2.1	**4.7**	4.0-5.5	**6.4**	5.3-7.5	**7.5**	5.6-9.4	**9.1**	6.1-12.1
**Hospitalization for SAH**	**1.3**	0.4-2.2	**1.7**	1.4-1.9	**3.0**	2.6-3.5	**5.0**	4.3-5.8	**6.6**	4.9-8.4	**9.0**	5.4-12.6
Male	**1.4**	-2.7-3.1	**1.4**	1.1-1.8	**2.3**	1.7-2.8	**3.9**	2.8-5.1	**6.2**	2.5-10.0	**8.6**	0.3-16.9
Female	**1.3**	0.3-2.3	**1.9**	1.5-2.3	**3.8**	3.2-4.4	**5.8**	4.8-6.8	**6.8**	5.0-8.7	**9.1**	5.2-13.0

**Hospitalization for DM**	**1.2**	0.0-2.4	**0.6**	0.4-0.7	**0.8**	0.6-1.0	**1.4**	1.0-1.9	**1.4**	0.6-2.2	**0.8**	0.3-1.3
Male	**2.0**	0.9-4.8	**0.6**	0.4-0.8	**0.7**	0.4-1.0	**1.6**	0.8-2.4	**0.8**	0.0-1.7	**0.5**	-0.4-1.4
Female	**0.6**	0.0-1.4	**0.5**	0.4-0.7	**0.9**	0.6-1.2	**1.3**	0.7-1.9	**1.7**	0.6-2.8	**0.9**	0.3-1.5
**Hospitalization (individuals with SAH and DM)***	**1.2**	-0.04-2.4	**0.7**	0.6-0.9	**1.5**	1.2-1.7	**2.5**	1.9-3.1	**3.5**	2.0-4.9	**4.2**	1.9-6.6
Male	**2.0**	-0.9-4.8	**0.7**	0.5-0.9	**0.9**	0.6-1.3	**2.4**	1.4-3.4	**2.0**	0.5-3.5	**1.4**	-0.07-2.9
Female	**0.6**	-0.05-1.4	**0.8**	0.6-1.0	**2.0**	1.5-2.4	**2.6**	1.9-3.3	**4.2**	2.2-6.2	**5.1**	2.1-8.1

*Utilization of the service for at least one of the diseases.

[1]Underweight: BMI < 18.5 Kg/m² / Normal weight: BMI = 18.5 - 24.9 Kg/m² / Overweight: BMI = 25.0 - 29.9 Kg/m² / Obesity I: BMI = 30.0 - 34.5 Kg/m² / Obesity II: BMI = 35.0 - 39.9 Kg/m² / Obesity III: BMI ≥ 40 Kg/m²

95%CI = 95% Confidence Interval / SAH = Systemic Arterial Hypertension / DM = Diabetes Mellitus
